# Acute Myocardial Infarction in a Patient with Two-Vessel Occlusion and a Large Lambl's Excrescence

**DOI:** 10.1155/2016/8370212

**Published:** 2016-11-22

**Authors:** Alfredo Pizzuti, Francesco Parisi, Luciano Mosso, Francesca Cali' Quaglia, Antonino Tomasello

**Affiliations:** ^1^Division of Cardiology, Mauriziano Hospital, Torino, Italy; ^2^Division of Cardiothoracic Surgery, Mauriziano Hospital, Torino, Italy; ^3^Unit of Pathology, Mauriziano Hospital, Torino, Italy

## Abstract

A 59-year-old man underwent an echocardiography study after myocardial infarction and it showed a thin, mobile mass attached to the aortic valve. A diagnosis of Lambl's excrescence (LE) was suspected. Coronary occlusion as a consequence of embolism of LE's material could not be excluded and the patient underwent surgical excision. Histology confirmed the diagnosis; however a differential diagnosis with papillary fibroelastoma could not be established because both of these structures are histologically indistinguishable. A brief survey of the literature is presented. Evidence-based recommendations for treatment have not been established yet.

## 1. Introduction

Lambl's excrescences (LE), described in 1856 [1], are thin, elongated, and hypermobile structures located at the coaptation point of cardiac valves' leaflets. They are almost exclusively seen on the left-sided valves with a large predominance of the aortic valve [2, 3]. Their prevalence in normal population is quite variable, from 0,7% to 38% [2–5]. These figures are higher in patients with stroke (from 22,5% to 47% [2, 5–7]). These figures are not modified by gender or age [2].

## 2. Case Report

**Figure 1:**
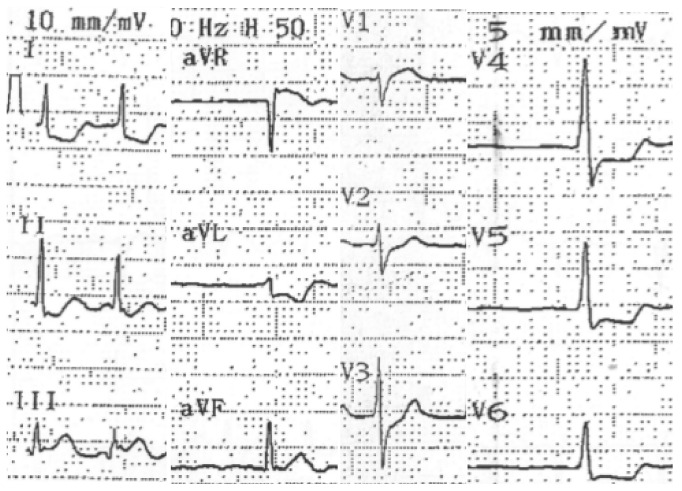
Electrocardiogram showing ST elevation in D3 and aVF leads and ST depression in leads exploring the lateral wall of the left ventricle.

**Figure 2:**
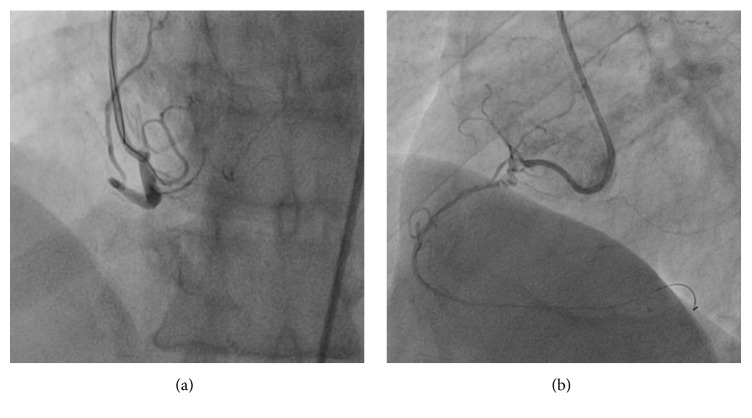
Coronary angiogram of the right coronary artery, in two projections, before (a) and after insertion of the guide-wire (b).

**Figure 3:**
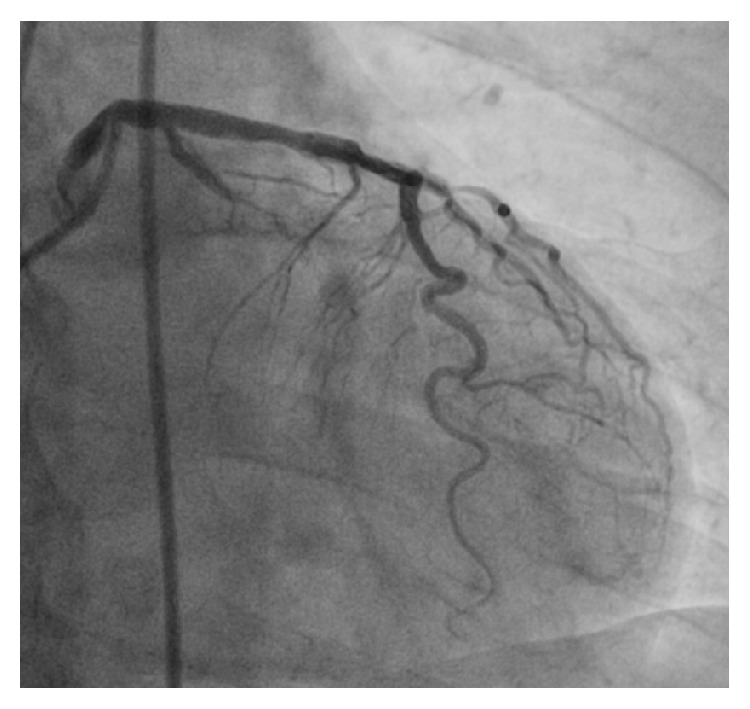
Coronary angiogram of the left coronary artery, showing almost totally occlusion of the circumflex artery.

**Figure 4:**
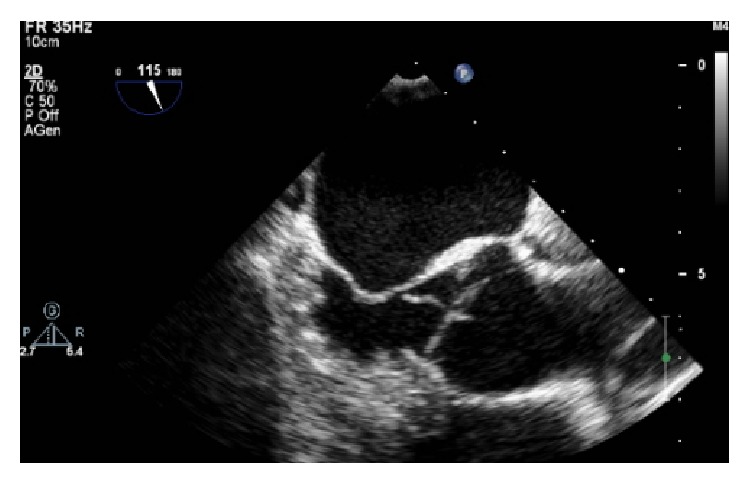
Transesophageal echocardiography. A linear structure arises from the line of closure of the aortic valve.

A 59-year-old man with hypertension and a negative history of cardiovascular or cerebrovascular events was admitted with symptoms of prolonged chest discomfort radiating to the left arm and sweating. The electrocardiogram showed mild ST-segment elevation in inferior leads and ST-segment depression in lateral leads (Figure 1). The cardiac troponin raised up to 3.75 *μ*g/L (normal value < 0.015). The coronary angiography showed occlusions of the proximal right (Figure 2) and the proximal left circumflex (Figure 3) coronary arteries. Unfortunately, thromboaspiration was not performed and multiple bare metal stents were implanted. After coronary revascularisation the patient underwent echocardiographic evaluation. The exam showed a normal left ventricular morphology and function (left ventricular ejection fraction = 0.58), mild mitral regurgitation, and trivial insufficiency of a normally shaped aortic valve with a thin, mobile echo attached to its ventricular side. Because there were no clinical signs of infectious endocarditis, a preliminary diagnosis of LE was formulated. The patient was referred to our Echo Laboratory for transesophageal echocardiogram in order to define a possible embolic source. A 22 mm long, filiform, hyper-mobile linear structure arising from the line of closure of the noncoronary cusp of the aortic valve and protruding through the valve during systole was identified (Figure 4, Video 1 in Supplementary Material available online at http://dx.doi.org/10.1155/2016/8370212). This finding was consistent with the presence of a large LE.

**Figure 5:**
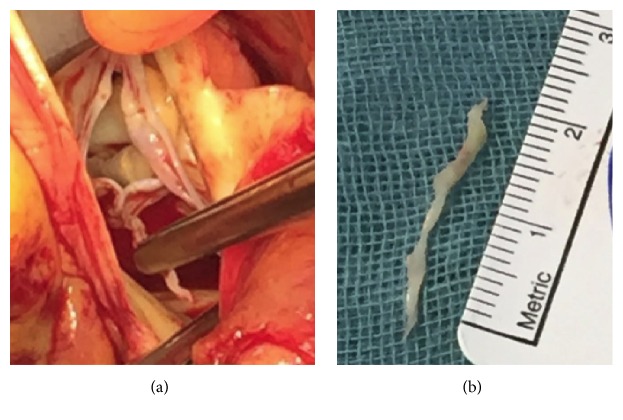
(a) Surgical view of the aortic valve. The excrescence is clamped and then (b) removed.

The hypothesis that the coronary occlusion was a consequence of embolism of LE's material could not be excluded. The patient was offered either life-long anticoagulation or surgical removal. After consultation with our institution's Heart Team (cardiologist, surgeon, and anesthesiologist) the patient decided to undergo cardiac surgery to remove the mass. The operation was performed in median sternotomy. A cardio pulmonary bypass (CPB) was established, the aorta was cross-clamped, and blood cardioplegia was infused. Access to aorta was made with standard transverse incision. The valve was exposed and the mass was easily removed from left aortic leaflet (Figure 5). Eventually, the aorta was closed. The cross-clamp time was 10 minutes and CPB time was 20 minutes. The procedure and postoperative recovery were uneventful.

**Figure 6:**
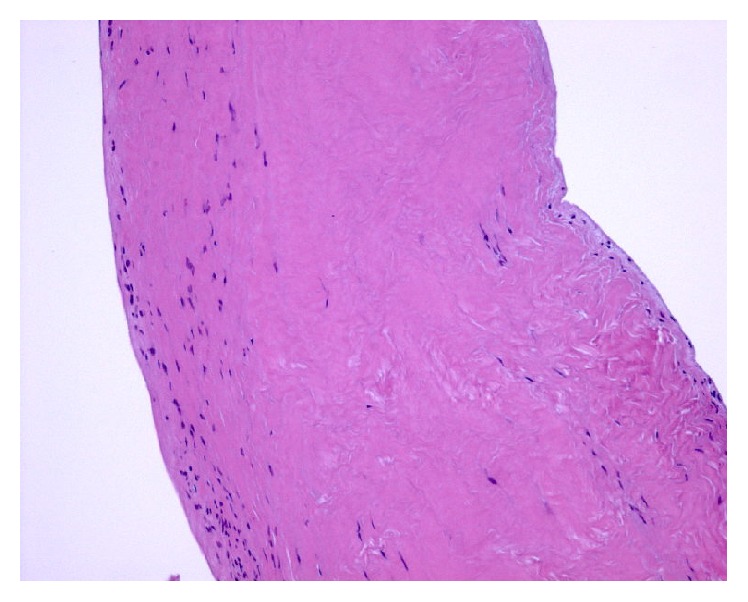
Histopathology: hematoxylin-eosin stain, magnification 100x.

**Figure 7 fig7:**
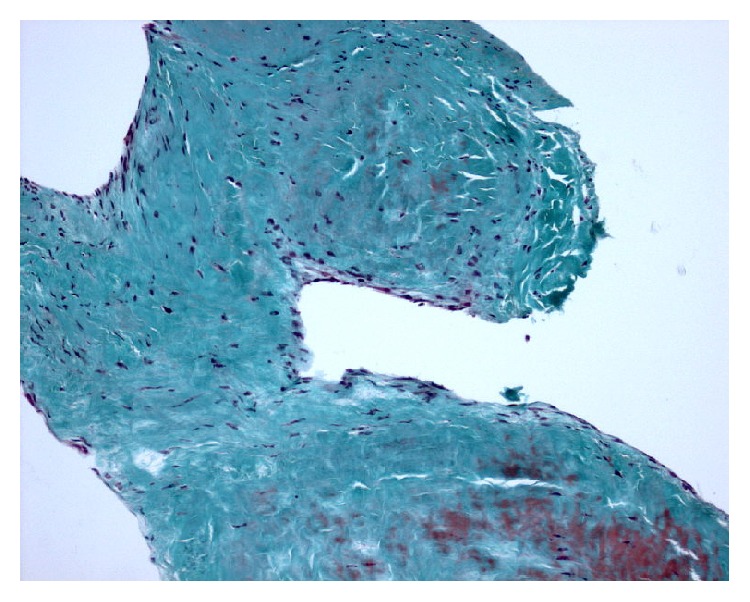
Histopathology: trichrome stain, magnification 100x.

**Figure 8:**
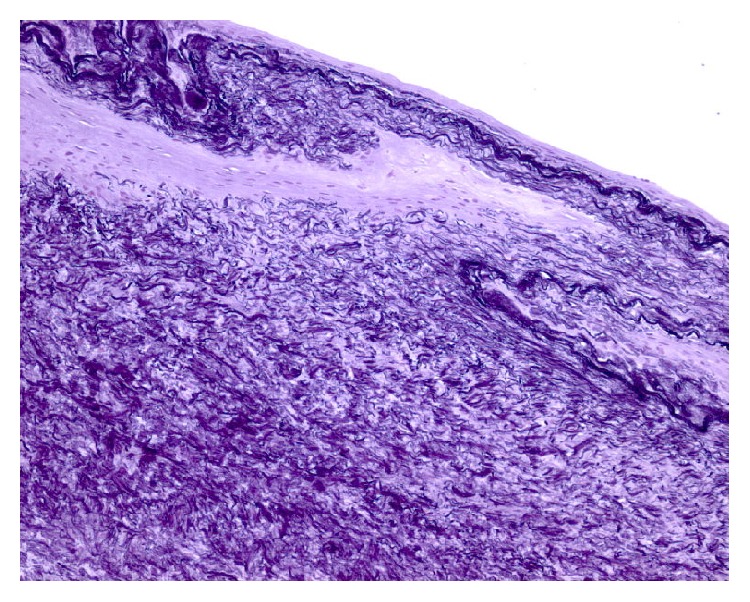
Histopathology: elastic fiber stain, magnification 100x.

The histopathology (Figures 6 [Fig fig7]–8) revealed a finger-like projection, similar to chordae tendinae, extending from the valve surface with a characteristic microscopic appearance: a matrix made of acellular collagen and a variable amount of elastic fibers with a surface covered by a single layer of endothelial cells.

## 3. Discussion

LE occur as singular or multiple strands and they are referred to as “giant” when multiple strands form a complex. In these cases LE should be differentiated from papillary fibroelastoma (PFE) (Table 1). Indeed, in the literature, a number of the cases are reported as giant LE and at the end appear to be PFE [8–11].

The distinction between LE and PFE is controversial both macroscopically and microscopically. Whereas LE arise from the line of closure of valve leaflets, most often in the Arantius nodules [12], PFE are usually attached to the downstream side of the valve, arising from the midportion of valve leaflets. They break out into fronds-like projections and can also be found on other areas of endocardium. PFE are larger and more gelatinous than Lambl's excrescences [13]. A typical aspect of both lesions, that is, a PFE attached by a thin, long pedicle to the line of closure of valve leaflets, has been seen in several cases [14]. Microscopically, LE and PFE are virtually identical; they both have a core of elastic connective tissue (fibrous body), surrounded by layers of fibrin and acid mucopolysaccharide matrix [15]. The classification of these two lesions may be artificial and based only on size and site [16]. According to some recent studies, LE and PFEs could be distinguished on the basis of the endothelial layers that are single in LE and multiple in PFE [17]. However, also this characteristic may be misleading, because a single layer of cells has also been described in PFE [15].

Even if a consensus is reached regarding a conservative strategy for silent, asymptomatic lesions accidentally discovered, the evidence-based recommendations have not been established yet. Giant LE may have increased thromboembolic potential [18–20]. On the other hand, also a thin, elongated LE may have a thromboembolic potential. Both Aggarwal and Leavitt [21] and Wu and colleagues [22] described two cases of women with recurrent strokes and thromboembolic infarction, despite adequate anticoagulant therapy, who were founded with a filamentous LE measuring, respectively, 25 mm and 20 mm in length. However, the LE's length reported in the literature is generally less than 20 mm, 4–16 mm in the old report of Roldan et al. [2] and 6 ± 2.4 mm in the large (150 patients) series of Leitman et al. [3]. Nevertheless, the embolic risk could not be directly related to LE's length, as we can read in Aziz and Baciewicz's report about a woman with repeated strokes and thin LE (1–4 mm long) on all the 3 leaflets of the aortic valve [23]. In a recent study focused on patients with Systemic Lupus Erythematosus Roldan and colleges reported that the LE's length was similar in patients with (11.9 ± 4.9 mm) and without (11.7 ± 3.5 mm) cerebrovascular disease and in controls (9.1 ± 3.4 mm) [24].

If the excrescence's length does not appear to be a strong predictor of events, its mobility could be as follows; in a large analysis of 725 cases of PFE by Gowda and colleagues the only independent predictor of tumor-related death or nonfatal embolisation was tumor mobility [13].

We found only one study that assessed the recurrence of stroke in presence of strands [4]; however all the patients included in this study were 60 or older, the strands were located only on the mitral valve, and the therapy was not randomized. Other information could be found in Homma and colleagues' study [7]; they demonstrate that when a stroke patient is treated medically the recurrence of stroke or death is the same between those with or without strands and that there is no difference, in terms of efficacy, between aspirin or warfarin's use. Unfortunately, because all the patients in those studies were medically treated, it was not possible to compare them with untreated patient, in order to assess the real efficacy of the treatment.

## 4. Conclusion

Valve excrescences are quite common on the aortic valves of normal subjects and patients. They can appear in form of thin, single, and elongated structures (LE), multiple strands (giant LE), or flower-like excrescence with multiple papillary fronds attached to the endocardium by a pedicle (PFE). All these structures are histologically indistinguishable. The vast majority of them are found incidentally in asymptomatic patients, but the clinical course could be devastating for someone.

Evidence-based recommendations for treatment have not been established yet. Symptomatic patients should be treated with surgical excision. If they are not suitable for surgery, they could be treated with long term anticoagulation. Asymptomatic patients could be switched to surgical approach, only if the lesion is mobile, because of the higher risk of death and nonfatal embolisation connected with the lesion mobility.

## Supplementary Material

Transesophageal echocardiography. A linear structure arises from the line of closure of the aortic valve protruding in the sinuses of Valsalva during systole.

## Figures and Tables

**Table 1:** Differences between LE, giant LE, and PFE.

	LE	Giant LE	PFE
Appearance	Thin, single, elongated structures	Complex forms resulting from adherence of multiple adjacent excrescences	Multiple papillary fronds attached to the endocardium by a short pedicle (sea-anemone-like)

Multiple	>90%	Always	Rarely

Dimension	The vast majority between 4 and 17 mm Very few described > 20 mm		Around 10 mm; up to 70 mm

Location	At sites of valve closure		More commonly from the midportion of the valve, away from the lines of closure; 23% on the endocardial nonvalvular surface

Histology	Core of elastic connective tissue surrounded by layers of fibrin and acid mucopolysaccharide matrix		
	Single layer of endocardial cells		Single or multiple layers of endocardial cells

Embolic risk	Low		High
